# Isolated patellofemoral osteoarthritis: A systematic review of treatment options using the GRADE approach

**DOI:** 10.3109/17453671003628756

**Published:** 2010-04-06

**Authors:** Hans-Peter W van Jonbergen, Rudolf W Poolman, Albert van Kampen

**Affiliations:** ^1^Department of Orthopedic Surgery, Deventer Hospital, Deventer; ^2^Department of Orthopedic Surgery, Onze Lieve Vrouwe Gasthuis, Amsterdam; ^3^Department of Orthopaedic Surgery and Orthopaedic Research Laboratory, Radboud University Nijmegen Medical Center, Nijmegenthe Netherlands

## Abstract

**Background and purpose** The optimal treatment for isolated patellofemoral osteoarthritis is unclear at present. We systematically reviewed the highest level of available evidence on the nonoperative and operative treatment of isolated patellofemoral osteoarthritis to develop an evidenced-based discussion of treatment options.

**Methods** A systematic computerized database search (Cochrane Database of Systematic Reviews, Cochrane Central Register of Controlled Trials, MEDLINE (PubMed), and EMBASE) was performed in March 2009. The quality of the studies was assessed independently by two authors using the Grading of Recommendations Assessment, Development and Evaluation (GRADE) approach.

**Results** We extracted data from 44 articles. The best available evidence for treatment of isolated patellofemoral osteoarthritis is sparse and of generally low methodological quality. Nonoperative treatment using physiotherapy (GRADE: high quality, weak recommendation for use), taping (GRADE: moderate quality, weak recommendation for use), or injection therapy (GRADE: very low quality, weak recommendation for use) may result in short-term relief. Joint-preserving surgical treatment may result in insufficient, unpredictable, or only short-term improvement (GRADE: low quality, weak recommendation against use). Total knee replacement with patellar resurfacing results in predictable and good, durable results (GRADE: low quality, weak recommendation for use). Outcome after patellofemoral arthroplasty in selected patients is good to excellent (GRADE: low quality, weak recommendation for use).

**Interpretation** Methodologically good quality comparative studies, preferably using a patient-relevant outcome instrument, are needed to establish the optimal treatment strategy for patients with isolated patellofemoral osteoarthritis.

## Introduction

A multitude of nonoperative and operative treatment options have been described for isolated patellofemoral osteoarthritis in the literature, but the optimal treatment is unclear at present. To develop an evidenced-based discussion of treatment options in isolated patellofemoral osteoarthritis, we reviewed the highest level of available evidence on the nonoperative and operative treatment of isolated patellofemoral osteoarthritis.

## Materials and methods

With use of the evidence-based cycle, we formulated 3 focused clinical questions with well-articulated Patient/Population (P), Intervention (I), Comparison (C), and Outcome (O) (PICO) elements (Poolman et al. 2007a). The questions were as follows. (1) In patients with isolated patellofemoral osteoarthritis (P), is physical therapy (I) better than no physical therapy (C) when assessed with a validated outcome measure (O)? (2) In patients with isolated patellofemoral osteoarthritis (P), is operative treatment (I) better than nonoperative treatment (C) when assessed with a validated outcome measure (O)? (3) In patients with isolated patellofemoral osteoarthritis (P), is patellofemoral arthroplasty (I) better than other operative treatment options (C) when assessed with a validated outcome measure (O)?

### Criteria for eligibility

We searched for studies that fulfilled certain inclusion criteria. Publications in the English, French, Dutch, or German language that describe the clinical outcome of nonoperative or operative treatments for isolated patellofemoral osteoarthritis in 10 or more patients were included. Publications reporting the results of treatment of patellofemoral pain syndrome without osteoarthritis were excluded, as were studies with incompletely described patient populations, insufficient descriptions of treatment, and studies lacking the use of validated or commonly used outcome measures.

### Study identification

Using the following search terms with Boolean operators ([femoropatell* OR femoro-patell* OR patell*] AND [osteoarthritis OR arthritis OR arthrosis]), we conducted the following searches:

1. Computerized database searches of: (a) the Cochrane Database of Systematic Reviews (2009, Issue 1); (b) the Cochrane Central Register of Controlled Trials (2009, Issue 1); (c) MEDLINE (PubMed) (1966 to 6 March 2009) using the “clinical queries” feature with a “broad search” for “therapy”; (d) EMBASE (1966 to 7 March 2009) using a search strategy with “Include sub-terms/derivatives” and “Record limits: Humans”.
2. Reviews of the bibliographies of eligible articles.

The systematic search was performed in March 2009 with adherence to the QUOROM statement and the MOOSE guidelines (Moher et al. 1999, Stroup et al. 2000). The search was performed in duplicate by one of the authors (HPWvJ) and a librarian. Authors of eligible studies were not contacted with regard to possible unpublished results.

### Evaluation of methodological quality

The quality of the studies included was assessed independently by two authors (HPWvJ, RWP) using the Grading of Recommendations Assessment, Development and Evaluation (GRADE) approach (www.gradeworkinggroup.org) (Atkins et al. 2004, Petrisor et al. 2006, Guyatt et al. 2008). Apart from describing the methodological quality of the studies (high, moderate, low, and very low), a strong or weak recommendation was given for or against the use of an intervention. A strong recommendation for using an intervention was given when the benefits clearly outweighed the risks for most if not all patients, with high-quality evidence supporting that recommendation. However, a strong recommendation against use may also be supported by studies of low-grade quality, such as case series that show serious adverse effects of the intervention (Poolman et al. 2007b). A weak recommendation for or against use of an intervention was given where the risks and benefits were more closely balanced or were more uncertain because of the low methodological quality of the supporting studies.

### Data abstraction

Relevant data regarding study design, study population, intervention, and outcome measures were extracted from the text, figures, and tables of the articles included.

## Results

44 studies, all of which were published as full journal articles, met the eligibility criteria and were included in this review (Figure and Table).

**Figure:**
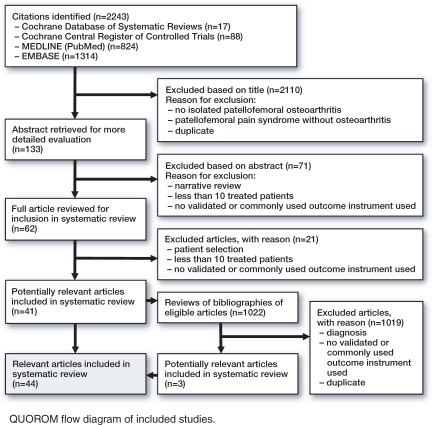
QUOROM flow diagram of included studies.

**Table:** GRADE evidence profile: nonoperative and operative treatment for isolated patellofemoral osteoarthritis

Quality assessment							Summary of findings				
A	B	C	D	E	F	G	H	I	J	K	L
Nonoperative treatment: Physiotherapy versus no physiotherapy											
1	RT	No	Yes	No	No	U	40	43	Knee pain at 5 months (–6.4 mm; 95% CI –15.3 to 2.4; p = 0.16) Increased quadriceps strength at 5 months (+11.7 Nm; 95% CI 4.5 to 19.0; p = 0.002) WOMAC at 5 months (-0.6; 95% CI -3.7 to 2.4; p = 0.68)	H	(+)
Nonoperative treatment: Taping											
1	RC	Yes (-1) ^b^	Yes	No	No	U	14	(14)	Neutral vs. medial taping: knee pain at 4 days (15.5 mm; 95% CI 2.4 to 28.6; p = 0.023) Neutral vs. lateral taping: knee pain at 4 days (-8.0 mm; 95%CI -22.5 to 6.5, p = 0.26)	M	(+)
Nonoperative treatment: Intra-articular injection											
1	O	Yes (-1) **^c^**	Yes	No	No	U	25	–		VL	(+)
Operative treatment: Arthroscopy											
2	RT	No	No	S (-1)	No	U	196	135	KSPS at 24 months (placebo 51.6±23.7; lavage 53.7±23,7; debridement 51.4±23.2; p = 0.64 and p = 0.96) WOMAC at 24 months (-23±605; 95% CI -208 to 161; p = 0.22)	M	–
Operative treatment: Chondroplasty, resection arthroplasty, and lateral facetectomy											
5	O	No	No	No	No	U	155	–		L	(+)
Operative treatment: Extensor mechanism alignment and lateral release											
7	O	No	No	No	No	U	224	–		L	(–)
Operative treatment: Patellectomy											
0											
Operative treatment: Total knee arthroplasty											
6	MO	No	No	No	No	U	271	–		L	(+)
Operative treatment: Patellofemoral arthroplasty											
24	SO	No	No	No	No	U	2,938	–		L	(+)
A. Number of studies											
B. Design											
RT: Randomized trial											
RC: Randomized crossover trial											
O: Observational											
MO: Matched case control, observational											
SO: Systematic review, observational											
C. Limitations											
No: No serious limitations											
Yes: Serious											
**^b^** patients not blinded, short follow-up.											
**^c^** pilot study, important heterogeneity in diagnosis.											
D. Inconsistency											
No: No serious inconsistency											
Yes: Only one study											
E. Indirectness											
No: No serious indirectness											
S: Some uncertainty about directness (-1), not specifically limited to isolated patellofemoral osteoarthritis.											
F. Imprecision											
No: No serious imprecision											
G. Publication bias											
U: Undetected											
H. Number of treated patients											
I. Number of controls											
J. Absolute effect and 95% confidence interval. KSPS: Knee Specific Pain Score.											
K. Quality											
H: High											
M: Moderate											
L: Low											
VL: Very low											
L. Recommendation											
(+): Weak for											
(–): Weak against											
–: Strong against											

Results relating to the 3 focused, patient-oriented clinical questions developed using PICO were as follows. 1 randomized controlled trial described the short-term outcome of physical therapy compared with no physical therapy (Quilty et al. 2003). We were unable to identify studies that directly compared the results of operative and nonoperative treatments. Also, no comparative studies were retrieved that directly compared the results of patellofemoral arthroplasty with the results of other operative treatment options.

Due to the heterogeneity of the study designs and outcome measures, a meta-analysis was not performed. The following review of the literature is therefore descriptive.

### Highest available evidence

1. Nonoperative treatment:
  - Physical therapy vs. no physical therapy: 1 randomized controlled trial (83 patients) (Quilty et al. 2003)
  - Taping: 1 randomized crossover trial (14 patients) (Cushnaghan et al. 1994)
  - Intra-articular injection: 1 prospective case series (25 patients) (Clarke et al. 2005)
2. Nonoperative vs. operative treatment:
  - No comparative studies identified.
3. Operative treatment:
  - Arthroscopy: 2 randomized controlled trials (165 and 168 patients) were included based on indirect evidence (Moseley et al. 2002, Kirkley et al. 2008)
  - Chondroplasty, resection-arthroplasty, and lateral facetectomy: 1 prospective case series (50 patients) (Becker et al. 2008) and 4 retrospective case series (11–63 patients) (Beltran 1987, Yercan et al. 2005, Spak and Teitge 2006, Paulos et al. 2008)
  - Extensor mechanism alignment and lateral release: 2 prospective case series (35 and 50 patients) (Alemdaroglu et al. 2008, Becker et al. 2008), 2 retrospective comparative studies (12 and 48 patients) (Weaver et al. 1991, Jacquot et al. 2004), and 3 retrospective case series (14–50 patients) (Aderinto and Cobb 2002, Kohn et al. 2004, Carofino and Fulkerson 2008)
  - Patellectomy: no studies met the inclusion criteria
  - Total knee arthroplasty: 2 matched case-control studies (94 and 54 patients) of total knee arthroplasty for isolated patellofemoral osteoarthritis compared with total knee arthroplasty for tri-compartmental osteoarthritis (Laskin and Van Steijn 1999, Meding et al. 2007), 1 prospective case series (24 patients) (Parvizi et al. 2001), and 3 retrospective case series (25–47 patients) (Mont et al. 2002, Dejour et al. 2004, Dalury 2005)
  - Patellofemoral arthroplasty: 3 systematic reviews of case series (538–812 patients) (Leadbetter et al. 2005, 2006, Becher et al. 2008), 5 prospective case series (15–240 patients) (Arnbjornsson and Ryd 1998, Tauro et al. 2001, Merchant 2004, Ackroyd and Chir 2005, Ackroyd et al. 2007), and 16 retrospective case series (12–65 patients) (Arciero and Toomey 1988, Cartier et al. 1990, Argenson et al. 1995, Krajca-Radcliffe and Coker 1996, Mertl et al. 1997, De Cloedt et al. 1999, Fink et al. 1999, de Winter et al. 2001, Smith et al. 2002, Kooijman et al. 2003, Board et al. 2004, Argenson et al. 2005, Cartier et al. 2005, Merchant 2005, Sisto and Sarin 2006, Gadeyne et al. 2008).

The available evidence together with background information from systematic reviews and other relevant sources was used for the following discussion of treatment options.

### Nonoperative treatment options

#### Physiotherapy.

Initially, patients with isolated patellofemoral osteoarthritis can be treated using a nonoperative approach such as activity modification, weight loss, and physiotherapy. 1 randomized controlled trial described the short-term outcome of a commonly used physiotherapy package (patellar taping, functional exercises, education, quadriceps strengthening exercises, postural advice, and education) compared with no physical therapy (Quilty et al. 2003). The physiotherapy intervention was delivered by a single physiotherapist in nine 30-minute sessions over 10 weeks, with advice to continue thereafter. The treatment group had a small reduction in pain and a substantial increase in the quadriceps strength of the index knee 10 weeks after treatment compared with the no-treatment group. After 12 months, no differences in patient-relevant outcome measures were noted between groups (Quilty et al. 2003). According to GRADE, the quality of this evidence is high, with a weak recommendation for use of the intervention.

#### Taping.

A randomized crossover trial using visual analog scale ratings for pain demonstrated a 25% reduction in knee pain when the patella was taped medially. However, each tape (medial, lateral, or neutral) was applied for only 4 days, with 3 days of no treatment between tape positions (Cushnaghan et al. 1994). According to GRADE, the quality of the evidence is moderate, with a weak recommendation for use of this intervention.

### Intra-articular injections/visco-supplementation

The clinical effect of intra-articular visco-supplementation with hylan G-F 20 (Synvisc; Genzyme Corporation, Cambridge, MA) was assessed in a non-randomized clinical trial with use of a patient-relevant outcome instrument. Pain upon stair climbing improved 4 weeks after the initial injection and the improvement was maintained to 26 and 52 weeks (Clarke et al. 2005). According to GRADE, the evidence is of very low quality, with a weak recommendation for use of this intervention.

### Operative treatment options

#### Arthroscopy.

We did not identify any studies describing the results of arthroscopic debridement of articular cartilage for patients with *isolated* patellofemoral osteoarthritis. However, we did include 2 methodologically sound randomized controlled trials, although they describe the results of arthroscopy in osteoarthritis of the knee, and were not specifically limited to isolated patellofemoral osteoarthritis (Moseley et al. 2002, Kirkley et al. 2008). No differences in outcome were found between surgical placebo treatment and arthroscopy, and between arthroscopy combined with physiotherapy as opposed to nonoperative treatment with physiotherapy only. Although these papers do not strictly describe the results of arthroscopic treatment for isolated patellofemoral osteoarthritis, indirect evidence is given. Based on these high-quality studies, arthroscopy is not recommended for osteoarthritis of the knee. In the case of indirect evidence, the GRADE group advises reducing the level of quality from high to moderate (Guyatt et al. 2008), with a strong recommendation against the use of this intervention.

#### Chondroplasty, resection-arthroplasty, and lateral facetectomy.

A retrospective case series in patients younger than 55 years of age showed that the use of fresh osteochondral allografts for patellofemoral arthritis resulted in relief of the arthritic condition, improved knee function, and delayed prosthetic knee replacement (Spak and Teitge 2006). A retrospective case series describing the results of en bloc removal of articular cartilage and subchondral bone showed that 20 of the 33 operated knees were pain-free after an average of 31-months of follow-up (Beltran 1987). Partial lateral facetectomy results in short-term improvement in pain scores with no or moderate improvement in function, as assessed with a patient-relevant outcome instrument (Yercan et al. 2005, Becker et al. 2008, Paulos et al. 2008). According to GRADE, the evidence is of low quality, with a weak recommendation for use of these interventions.

#### Extensor mechanism alignment and lateral release.

Anterior displacement of the tibial tuberosity reduces the contact forces, but not necessarily the stress on the patellofemoral joint (Lewallen et al. 1990). Anteromedialization, which translates the contact area medially, results in relief of the lateral facet which could theoretically reduce pain. Retrospective case series evaluating the 2- to 6-year results of anteromedial transfer of the tibial tuberosity combined with lateral retinacular release have demonstrated an improvement in outcome measures with reduced pain (Weaver et al. 1991, Kohn et al. 2004, Carofino and Fulkerson 2008). Total loss of cartilage or absence of lateralization are contraindications to the Fulkerson procedure (Steimer and Kohn 2007). Compared with medialization with vastus medialis obliquus shortening, anterior displacement and lateral facetectomy both result in improved knee function (Jacquot et al. 2004). However, the number of complications associated with the Maquet anterior displacement is high (Kadambande et al. 2004). Combined partial lateral facetectomy, lateral release, and medialization of the tibial tubercle result in incomplete improvement of symptoms as assessed with a patient-relevant outcome instrument (Becker et al. 2008). In a large number of patients, isolated arthroscopic lateral retinacular release results in reduction of pain rather than resolution (Aderinto and Cobb 2002, Alemdaroglu et al. 2008). In evaluating the results, a patient-relevant outcome instrument was used. According to GRADE, the evidence is of low quality, with a weak recommendation against use of these interventions.

#### Total knee arthroplasty.

Total knee replacement with patellar resurfacing gives satisfactory 5- to 7-year results in patients with isolated patellofemoral osteoarthritis (Laskin and Van Steijn 1999, Parvizi et al. 2001, Mont et al. 2002, Dejour et al. 2004, Dalury 2005, Meding et al. 2007). These results are similar to those achieved after total knee arthroplasty with patellar resurfacing for femorotibial osteoarthritis (Laskin and Van Steijn 1999, Meding et al. 2007). However, up to one-fifth of patients have reported anterior knee pain after total knee replacement (Laskin and Van Steijn 1999, Parvizi et al. 2001, Mont et al. 2002, Meding et al. 2007). As with total knee arthroplasty for tricompartmental osteoarthritis, it remains unclear whether patellar resurfacing results in better outcomes in isolated patellofemoral osteoarthritis (Thompson et al. 2001). Because of its relationship with patellofemoral instability, total knee arthroplasty in patients with isolated patellofemoral osteoarthritis is a technically more demanding procedure (Laskin and Van Steijn 1999, Parvizi et al. 2001, Mont et al. 2002, Saleh et al. 2005). According to GRADE, the evidence is of low quality, with a weak recommendation for use of this intervention.

#### Patellofemoral arthroplasty.

In patellofemoral arthroplasty, the femorotibial compartments with cruciate ligaments and menisci are spared, which probably allows preservation of physiological femorotibial joint mechanics. The clinical results reported are related to prosthetic design, surgical technique, patient selection and indication, and length of follow-up, and have shown good to excellent 3- to 17-year results in two-thirds of patients to all of them (Arciero and Toomey 1988, Cartier et al. 1990, Argenson et al. 1995, Krajca-Radcliffe and Coker 1996, Mertl et al. 1997, Arnbjornsson and Ryd 1998, de Winter et al. 2001, Smith et al. 2002, Kooijman et al. 2003, Merchant 2004, 2005, Ackroyd and Chir 2005, Cartier et al. 2005, Sisto and Sarin 2006, Ackroyd et al. 2007, Gadeyne et al. 2008). Progression of femorotibial osteoarthritis, malposition of the prosthesis, and wear or loosening may result in failure of the patellofemoral arthroplasty (Leadbetter et al. 2005). Development of painful femorotibial osteoarthritis is the most important non-prosthetic-related reason for conversion to total knee arthroplasty. Conversion rates of 1 in 5 have been reported after an average of 7 to 16 years (Kooijman et al. 2003, Argenson et al. 2005). It remains unclear which patients are at risk of developing femorotibial osteoarthritis (Leadbetter et al. 2005). Recently, the results of revision to total knee arthroplasty for progression of femorotibial osteoarthritis or malposition was described (Lonner et al. 2006). Clinical outcome as assessed by the Knee Society score (KSS) improved after revision. Patellofemoral arthroplasty does not have a negative effect on the outcome of later total knee arthroplasty (van Jonbergen et al. 2009). According to GRADE, the evidence is of low quality, with a weak recommendation for use of this intervention.

## Discussion

Several nonoperative and operative treatment options for isolated patellofemoral osteoarthritis have been described. At present, there are no publications describing the outcome of nonoperative treatment after 1 year. A multitude of studies of generally low methodological quality have reported the short- and long-term results of surgical management. Despite these limitations, we present the following treatment recommendations based on the best available evidence.

Nonoperative treatment using physical therapy (GRADE: high quality, weak recommendation for use), taping (GRADE: moderate quality, weak recommendation for use), or injection therapy (GRADE: very low quality, weak recommendation for use) may result in short-term relief. Joint-preserving surgical treatment may result in insufficient, unpredictable, or only short-term improvement (GRADE: low quality, weak recommendation against use). Total knee replacement with patellar resurfacing results in predictable and durable good results (GRADE: low quality, weak recommendation for use). However, for a degenerative disease involving only one compartment, it is probably too aggressive. Outcome after patellofemoral arthroplasty in selected patients is good to excellent (GRADE: low quality, weak recommendation for use). Total knee replacement can be performed later if painful femorotibial osteoarthritis develops.

### Strengths and limitations of this review

Our study is the first systematic review to use both well-articulated patient-oriented clinical questions (PICO) and an evaluation using the GRADE approach in order to obtain an evidenced-based discussion of nonoperative and operative treatment options in isolated patellofemoral osteoarthritis. However, our study has some limitations that should be considered. First, there is always the possibility that we failed to identify some studies, although a comprehensive search strategy was used including visually searching the reference lists of all eligible articles. Secondly, our aim was to evaluate the best evidence on the treatment of patellofemoral osteoarthritis, and therefore we did not include chondromalacia in our search strategy. Because there is currently no consensus on the diagnostic criteria of patellofemoral osteoarthritis, it is possible that we included studies with important heterogeneity among the degree of osteoarthritis and clinical complaints.

### Limitations of primary research

This systematic review shows that the current best available evidence for treatment of isolated patellofemoral osteoarthritis is sparse and generally of low methodological quality. The lack of randomized, controlled studies may result in substantial selection bias. Also, comparison of the results of different treatments is hampered by the extensive heterogeneity among the outcome instruments used. Only 4 of the 44 studies included employed a patient-relevant outcome instrument such as the WOMAC osteoarthritis index in evaluating the results of treatment (Quilty et al. 2003, Clarke et al. 2005, Alemdaroglu et al. 2008, Becker et al. 2008).

### Implications for future research

Methodologically good-quality studies, preferably evaluating results with a validated patient-relevant outcome measure such as the KOOS or WOMAC (Paxton and Fithian 2005), are needed to establish the optimal treatment strategy for patients with isolated patellofemoral osteoarthritis. Ideally, such studies should compare the results of commonly advocated methods of nonoperative and operative treatments.

### Conclusion

The results of this systematic review show that the best available evidence for nonoperative and operative treatment options for patients with isolated patellofemoral osteoarthritis is sparse and of low methodological quality. Presently, there is no convincing evidence that one specific treatment modality is superior to another in terms of better outcomes.
